# Efficiency in National Health

**Published:** 1921-02-19

**Authors:** 


### The Hospital

The Workers' Newspaper of Administrative Medicine and Institutional Life. Administration. National Insurance and Health.

No. 1811, Vol. LXIX. SATURDAY, FEBRUARY 19, 1921. PRICE TWOPENCE

### EFFICIENCY IN NATIONAL HEALTH.

Every person, whatsoever his calling, who has visited the Olympia this week must now have a firm conviction that not only is efficiency the pathway to prosperity, but also that opportunity for national cultivation of this attribute is well-nigh unlimited. On every hand he saw new methods of overcoming difficulties, for years considered insuperable, and suffered as long in relatively lethargic silence. Did he attend any one of the many conferences, the faults of the past and their remedies in the future must still be occupying no small section of his daily thoughts.

But in an exhibition covering so wide a field one of the outstanding points from which we derive satisfaction is the importance which the practical mind of to-day is attributing to national health. In our pages this week we give a brief review of some appropriate exhibits, and it is, indeed, remarkable how few of all these aids to efficiency do not directly or indirectly benefit the health of both the worker and the nation. In such profusion it is however excusable that we should have our favourites. A number of eminent medical men, whose first-hand experience the nation has regrettably few opportunities of hearing from the public platform, have given advice simple, authoritative, and correspondingly invaluable. Two famous London hospitals have demonstrated to the people the importance of research in medicine. Research is a subject so far truly appreciated only by the few because, in practical form, it has never been brought to the notice of the many. Now, however, the pioneer attempt has been made, and we can only trust that thousands of people have seen and appreciated the efforts that both the Middlesex and St. Mary's hospitals have made in furthering a vital section of public education.

On another point, too, we should express our satisfaction. Although both these hospitals have, at the moment, their own special financial appeal, yet the fact is in neither case overstressed. The labours and the responsibilities instanced by their exhibits are put forward as the heritage of every great hospital and medical school without exception. The literature and pamphlets obtainable at the stands maintain the same note, and in the words of the Earl of Athlone, an attempt is here made simply to explain how the subject intimately affects the interests of the whole community. It is not primarily an appeal to charity, though it is self-evident that research is costly and funds are needed.

In any case, the layman is brought to an almost abrupt realisation that there are thousands of factors, highly skilled workers, and processes involved in the care of the nation's health; vastly more than the majority have ever pictured in their most imaginative moments. To realise it, one has but to talk with them as they gaze at the commonest of laboratory processes. In all the popular but obscuring discussions on record cards, panel systems, unknown and terrifying diseases, and the threat of bureaucracy, is it surprising that the public have failed to realise that in many ways popular journalism has rarely taken them below the surface of things? We sincerely trust that many heard and appreciated the unanimous congratulations voted at the end of the series of health conferences to the Ministry concerned. Admittedly, there were resolutions urging still further propaganda on sundry vital subjects, but, by all means, let us appreciate what has already been accomplished.

From the purely hospital point of view it is perhaps a matter for regret that only the research and laboratory departments are actually exhibited. Efficiency is essentially the keynote of successful institutional work. In the presence of adequate financial support the attribute could hardly be better illustrated than by any of the inner workings of a first-class voluntary hospital. Every part of it, the wards, the out-patient departments, the operating-threatres, all might be displayed to convey their equally impressive public message. But, perhaps, this development will come another year. The success of the present attempt makes such expansion well worth while.

There is, however, a further exhibit, or rather a great series of them, which should appeal to every person in the country. By this we refer to the Ministry of Labour's demonstration of the work that is being done to instruct war-disabled men in skilled trades fitted to their disabilities. A training department having been established, a number of instructional factories organised throughout the country, and maintenance allowances made, arrangements are now complete whereby the men can finish their training in appropriate workshops on terms depending on the trade which they enter. Against certain quite unjustifiable opposition to the active industrial enlistment of these recruits, we would here express the strongest protest. Best to appreciate the real situation, we would urge that every interested person should see for himself at Olympia what these men have done and their potential value to the country. 24,000 of the disabled are already trained, besides 11,000 previously instructed through the Ministry of Pensions, and 25,000 more are in training at the present moment. The men have done their share. The public can do theirs. As employers, they can take them into their factories. As trade unionists, they can facilitate their progress in the shops. These men can well maintain the best "traditions of the trade," if the people will maintain our national traditions for Justice.

